# The effect of probiotic supplementation in older adults with mild cognitive impairment and Alzheimer’s disease: A triple‐blind, randomized, placebo‐controlled clinical trial

**DOI:** 10.1002/alz70861_108842

**Published:** 2025-12-23

**Authors:** Josiane Budni, Eduarda Behenck Medeiros, Gabriela Piovesan Fenilli, Adrielly Vargas Lidio, Débora Dagostin Casagrande, Gabriela Serafim Keller, Carolina Speling Mendes Souza, Luize do Canto Velho, Bruno Kluwe‐Schiavon, Tatiana Barichello

**Affiliations:** ^1^ University of Southern Santa Catarina (UNESC), Criciúma Brazil; ^2^ University of Southern Santa Catarina (UNESC), Criciuma, SC Brazil; ^3^ University of Southern Santa Catarina, Criciúma Brazil; ^4^ The University of Texas Health Science Center at Houston, Houston, TX USA; ^5^ Universidade do Extremo Sul Catarinense, Criciuma, SC Brazil

## Abstract

**Background:**

Population is aging, bringing with it changes associated with senescence and senility, including a progressive decline in functional capacities. Alzheimer’s disease (AD) is the most common form of dementia affecting the elderly population. In addition to observable impairments, there are also alterations that directly impact the central nervous system (CNS), such as increased neuroinflammation. While the inflammatory processes related to aging are well documented, recent research has focused on understanding the relationship between dysbiosis and changes in the gut microbiota and how these can influence CNS function. Based on this context, the present study aimed to evaluate the effects of probiotic supplementation on cognitive, inflammatory, and neurotrophic parameters in older adults as cognitively healthy, with mild cognitive impairment (MCI), or Alzheimer’s disease (AD).

**Method:**

This study was approved under protocol number 7,041,292 and registered in the Brazilian Registry of Clinical Trials (RBR‐9gccx5c). This is a placebo‐controlled, triple‐blind, randomized clinical trial conducted with individuals aged 60 years and older, of both sexes, residing in long‐term care facilities. Initially, participants underwent a sociodemographic assessment, cognitive testing, and biological sample collection. Following this, they were randomly assigned to receive either placebo capsules or a probiotic blend. After 12 weeks of treatment, all assessments were repeated to compare pre‐ (T1) and post‐treatment (T2).

**Result:**

The final sample included 53 individuals (16 controls, 18 with MCI, and 19 with AD). Participants in the AD group were significantly older and had lower educational levels. When comparing cognitive test results, the control group performed significantly better than participants with MCI or AD. In terms of cytokine levels, an increase in IL‐1β, IL‐6, and TGF‐β, and a decrease in IL‐4, IL‐10, and BDNF were observed at T2 compared to T1. Additionally, significant interactions between time and treatment were found for TNF‐α, GDNF, and NGF levels: TNF‐α and GDNF increased in the control group receiving probiotics, while NGF increased in the MCI group.

**Conclusion:**

These findings suggest that probiotic supplementation influenced levels of TNF‐α, GDNF, and NGF, underscoring the importance of clinical research into the role of gut microbiota in maintaining brain health.

